# Supplementary material for: MRI signatures of cortical microstructure in human development align with oligodendrocyte cell-type expression
Source: Nat Commun. 2025 Apr 7;16:3317. doi: 10.1038/s41467-025-58604-w (PMC11977195; doi:10.1038/s41467-025-58604-w)
Supplement: Supplementary file 1 — Supplementary Information [file 41467_2025_58604_MOESM1_ESM.pdf]

Supplementary materials

Figures

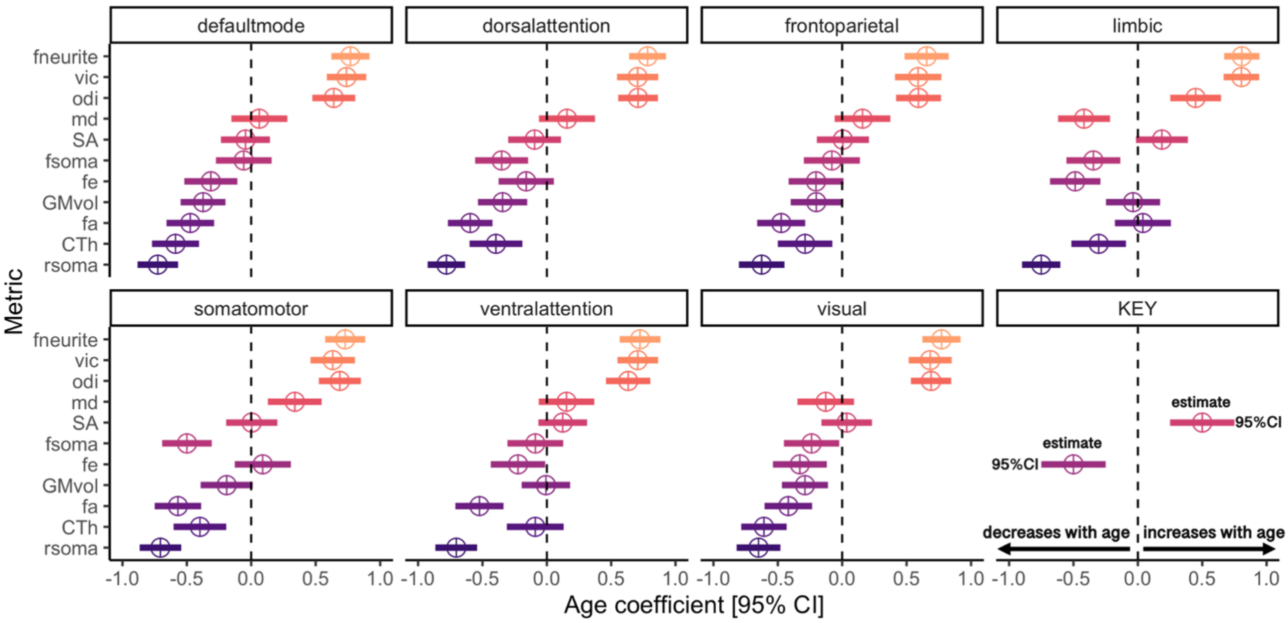

Figure S1: Direction and magnitude of age-relationships for commonly investigated morphological and microstructural measures. Multiple linear models investigating age-relationships in commonly investigated white matter metrics were performed in N=88 participants, adjusting for sex. Standardised coefficients from these linear models are shown for each metric, with error bars representing 95% confidence intervals. Significant age associations do not cross 0 the 95% confidence interval (CI). Metrics with a crossbar positioned left of 0 suggest a significantly negative relationship with age, such as Rsoma and cortical thickness. Metrics with a positive crossbar positioned suggest a significantly positive relationship with age, such as fneurite, vic and odi. Abbreviations: CTh: Cortical thickness; fa: fractional anisotropy; fe: extracellular signal fraction; fneurite: neurite signal fraction; fsoma: soma signal fraction; GMvol: Grey matter volume; md: mean diffusivity; odi: orientation dispersion index; rsoma: apparent soma radius, in μm; SA: surface area; vic: intracellular volume fraction.

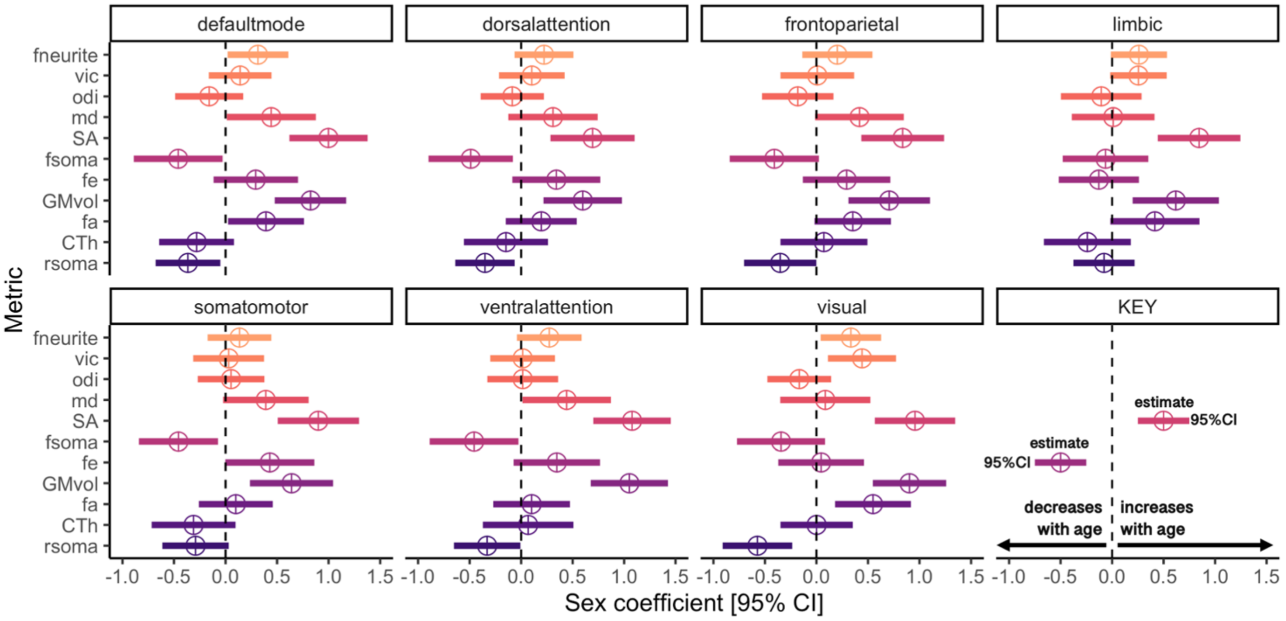

Figure S2: Direction and magnitude of sex differences for commonly investigated morphological and microstructural measures. Multiple linear models investigating sex differences in commonly investigated white matter metrics were performed in N=88 participants, adjusting for age. Coefficients from these linear models are shown for each metric, with error bars representing 95% confidence intervals. Significant sex differences do not cross 0 the 95% confidence interval (CI). Metrics with a crossbar positioned left of 0 suggest higher values in females, such as f_{soma}. Metrics with a positive crossbar positioned suggest higher values in males, such as grey matter volume and surface area. Abbreviations: CTh: Cortical thickness; fa: fractional anisotropy; fe: extracellular signal fraction; fneurite: neurite signal fraction; fsoma: soma signal fraction; GMvol: Grey matter volume; md: mean diffusivity; odi: orientation dispersion index; rsoma: apparent soma radius, in µm; SA: surface area; vic: intracellular volume fraction.

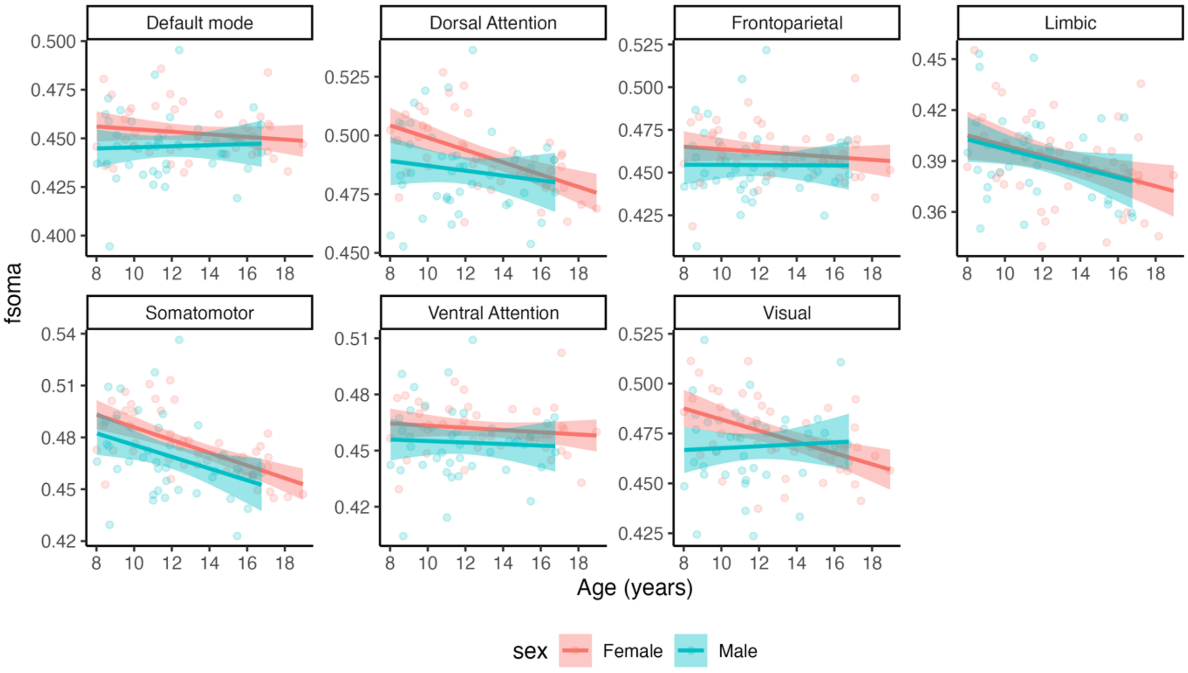

Figure S3: Sex differences in f_{soma} investigated in N=88 participants On average, females had higher soma signal fraction (f_{soma}) compared with males, but none of these associations were statistically significant. Curves represented as mean trajectory with 95% confidence interval bounds.

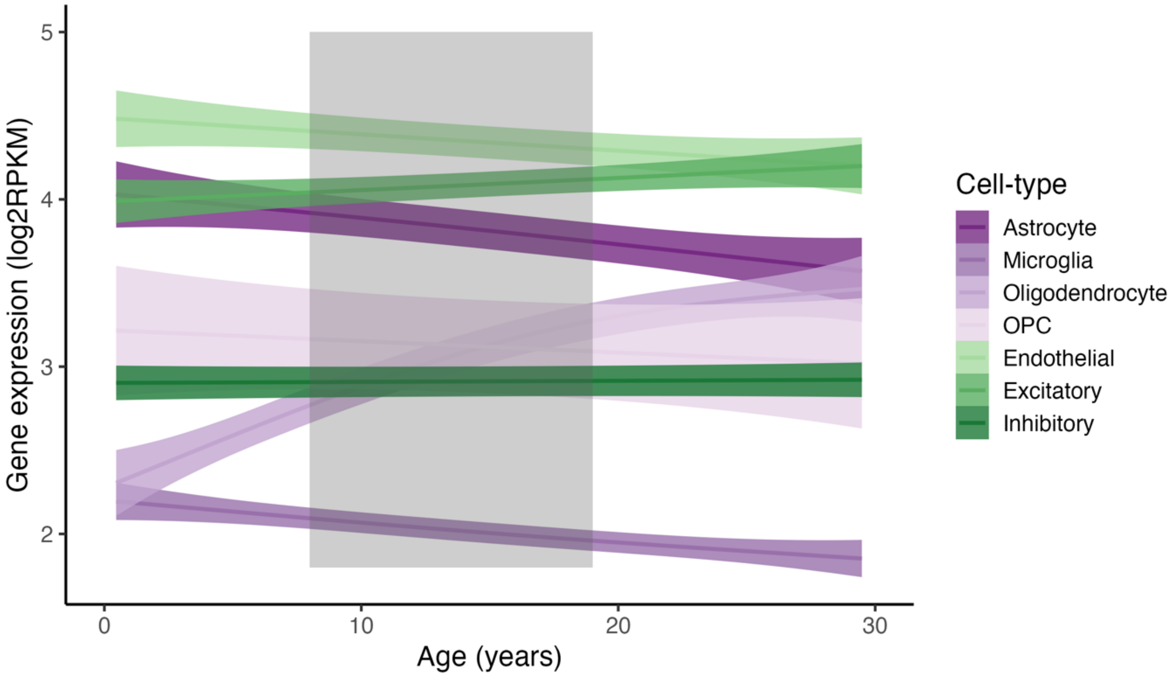

Figure S4: Developmental trajectories of genes in the PsychENCODE dataset expressed by cell class. Data shown for samples aged 0-30 years, expressed in log_2-reads-per-kilobase of transcript per million (log_2RPKM). Shaded area represents the age window of our in vivo MRI data set (8-19 years). Gene expression was relatively higher for endothelial cells, inhibitory neurons, and astrocytes. Gene expression was relatively low for microglia. Curves represented as mean trajectory with 95% confidence interval bounds. Source data to generate this graph are provided in source data file for Fig 3b. Abbreviations: OPC: oligodendrocyte precursor cell.

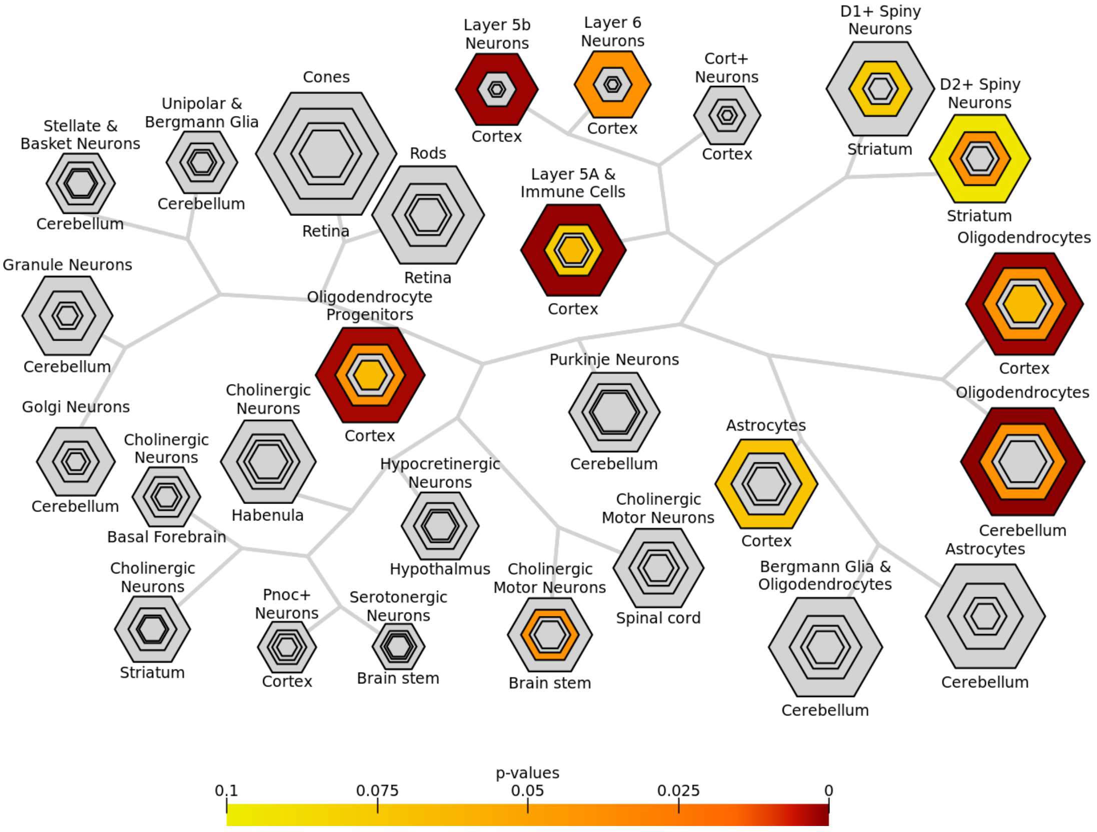

Figure S5: Cell-type Specific Expression Analysis (CSEA) of developmentally sensitive genes. Of the 467 age-related genes we identified (see appended gene list in 13.1.1), 407 of these genes could be mapped to specific cell types in the CSEA database (Xu, Wells [1]; colour bar indicates p_{FDR}<.05). Hexagon size scales with enrichment (overlap) of age-related genes in genes expressed by each cell type. Concentric rings indicate cell specificity thresholds, with inner rings indicating high cell specificity. Rings are coloured by p-value threshold (e.g., dark red indicates significant associations at p_{FDR}<.001). Note: the CSEA database uses mouse transcriptomic profiling to identify cell-specific genesets. Figure created using https://doughertytools.wustl.edu/CSEAtool.html.

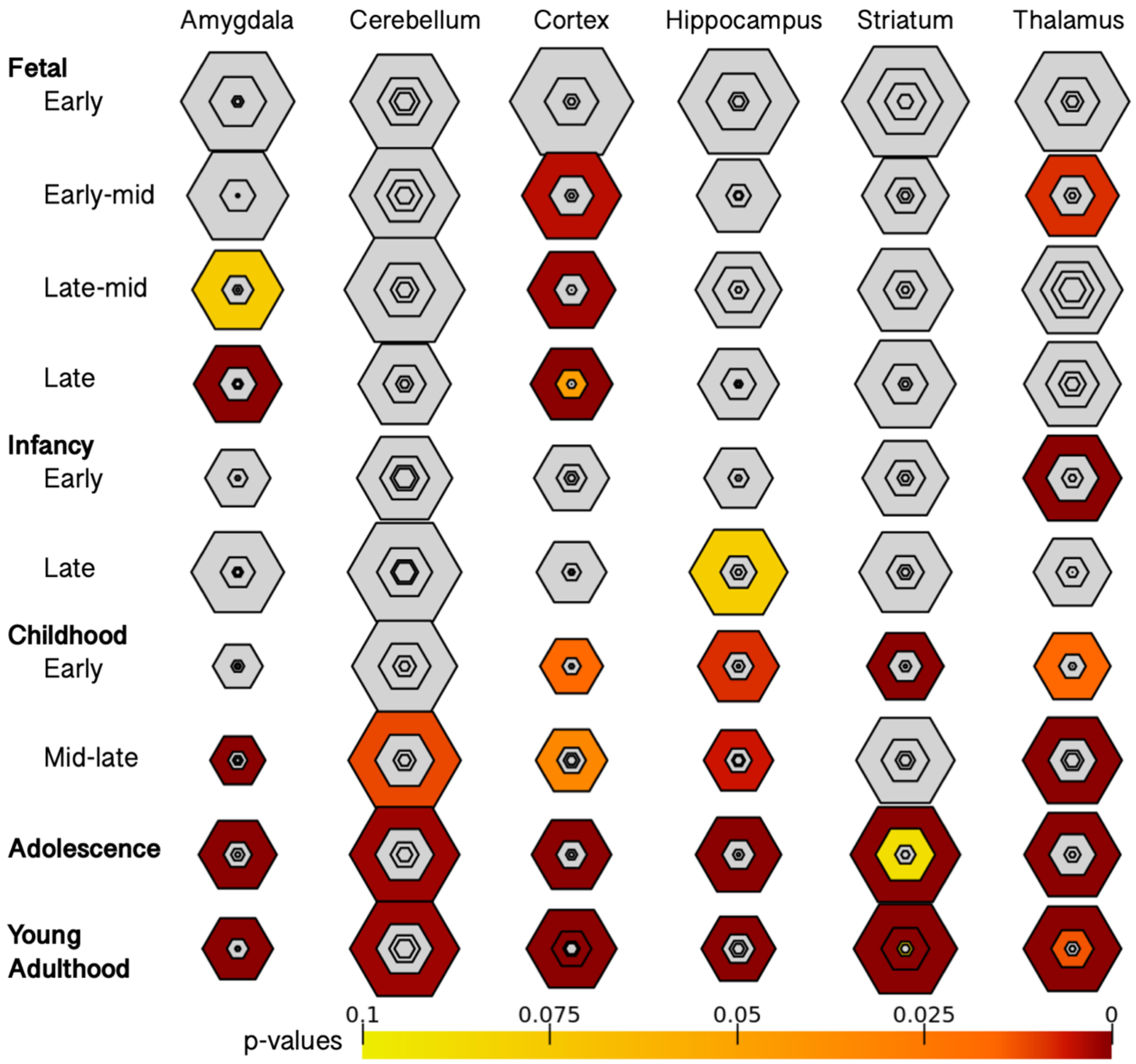

Figure S6: Specific Expression Analysis (SEA) of age-related genes across brain region and development. Of the 467 genes we identified that were significantly correlated with age (see appended gene list in 13.1.1), a subset of these genes corresponded to specific developmental stages (n=446 genes) in the CSEA[1] database. Colour bar indicates p_{FDR}<.05. Hexagon size scales with enrichment (overlap) of age-related genes in genes expressed by each cell type. Concentric rings indicate cell specificity thresholds, with inner rings indicating high cell specificity. Note: the CSEA database uses mouse transcriptomic profiling to identify cell-specific genesets. Figure created using https://doughertytools.wustl.edu/CSEAtool.html.

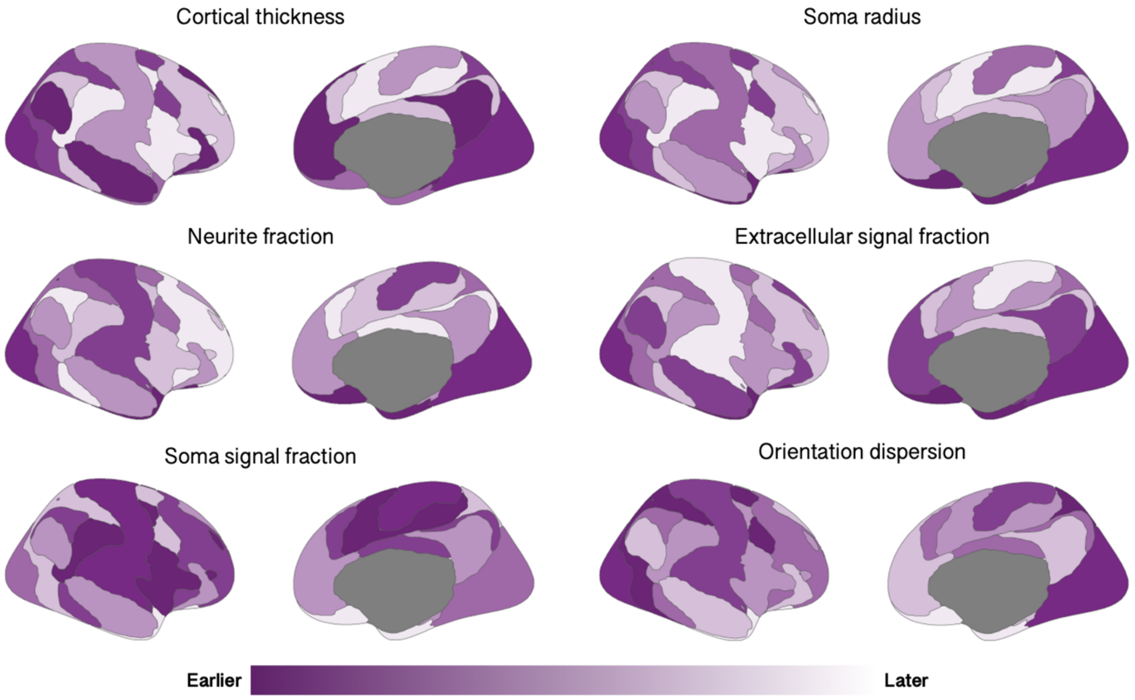

Figure S7: Peak maturation of cortical microstructure and morphology. Darker colours indicate regions reaching earlier maturation. Visual, limbic and somatomotor regions are generally reaching peak maturation earlier, particularly in neurite fraction and orientation dispersion. Colour scale visualised over Yeo7 atlas. Figure created using the ggseg package in R (ggseg.github.io/ggseg/).

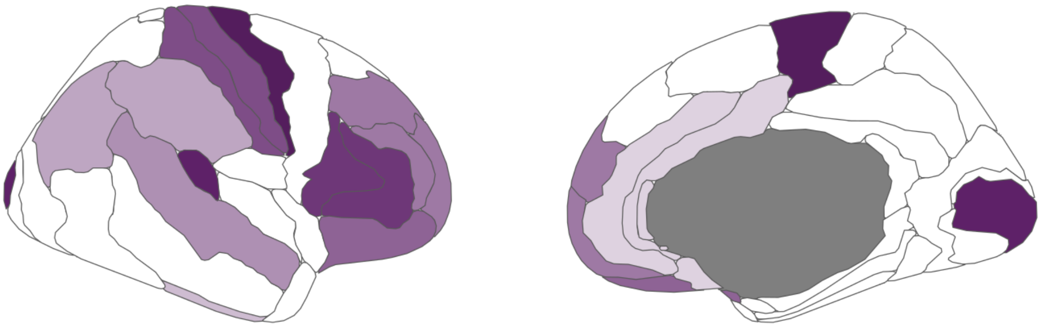

Figure S8: Spatial patterns of peak gene expression of oligodendrocyte cell-types. Figure created using the ggseg package in R (ggseg.github.io/ggseg/).

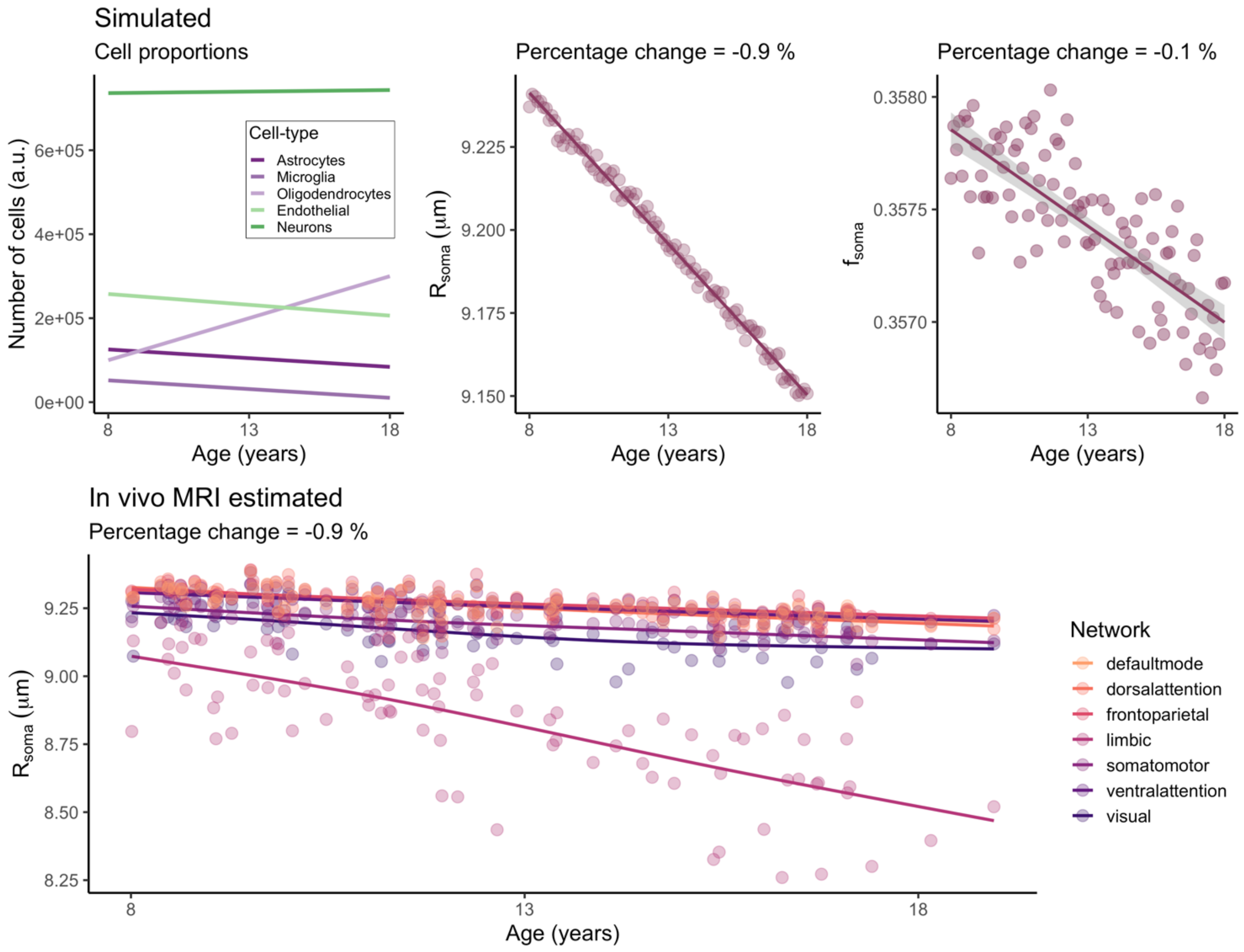

Figure S9: Cell type composition changes based on the actual expected distribution of cell body radii within a voxel based on realistic cell counts outlined in Keller, Erö [2]. Assuming gene expression patterns closely map on to cell numbers, simulated R_{soma} based on known cell densities and sizes shows a decrease in R_{soma} over age (1% decrease) at the same rate to in vivo estimates (1% decrease). Curves represented as mean trajectory with 95% confidence interval bounds. Abbreviations: f_{soma}: soma signal fraction; R_{soma}: apparent soma radius, in µm.

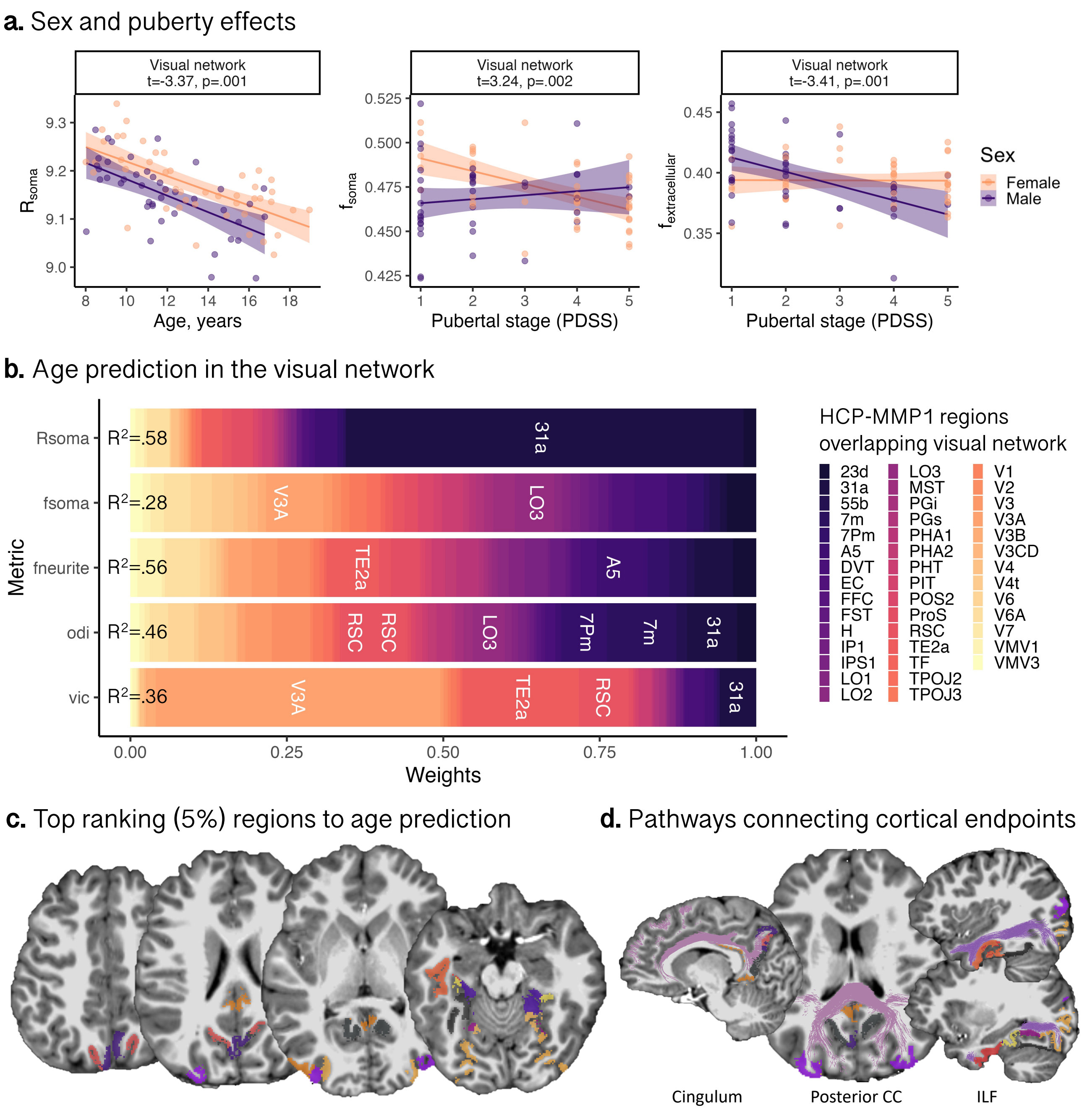

Figure S10: Developmental patterns of microstructure in the visual network. Analysis performed in N=88 participants. (a) Sex differences in R_{soma}, and sex by puberty interactions for f_{soma} and $f_{extracellular}$ signal fractions. (b) Feature importance of regions overlapping visual network [3] to brain age estimation; top ranking regions with a weighting >5% (width of coloured bin) are in white text and accuracy of prediction model is represented (as R^2) on the leftmost point of the bar plot. (c) Top ranking regions overlaid on a representative participant, coloured by labels in (b). (d) White matter pathways derived from tractography connecting cortical endpoints identified in age prediction analysis, such as the cingulum, posterior corpus callosum (CC) and inferior longitudinal fasciculus (ILF) which traverse regions in (c). Abbreviations: $f_{neurite}$: neurite signal fraction; f_{soma}: soma signal fraction; odi: orientation dispersion index; R_{soma}: apparent soma radius, in μm; vic: intracellular volume fraction;

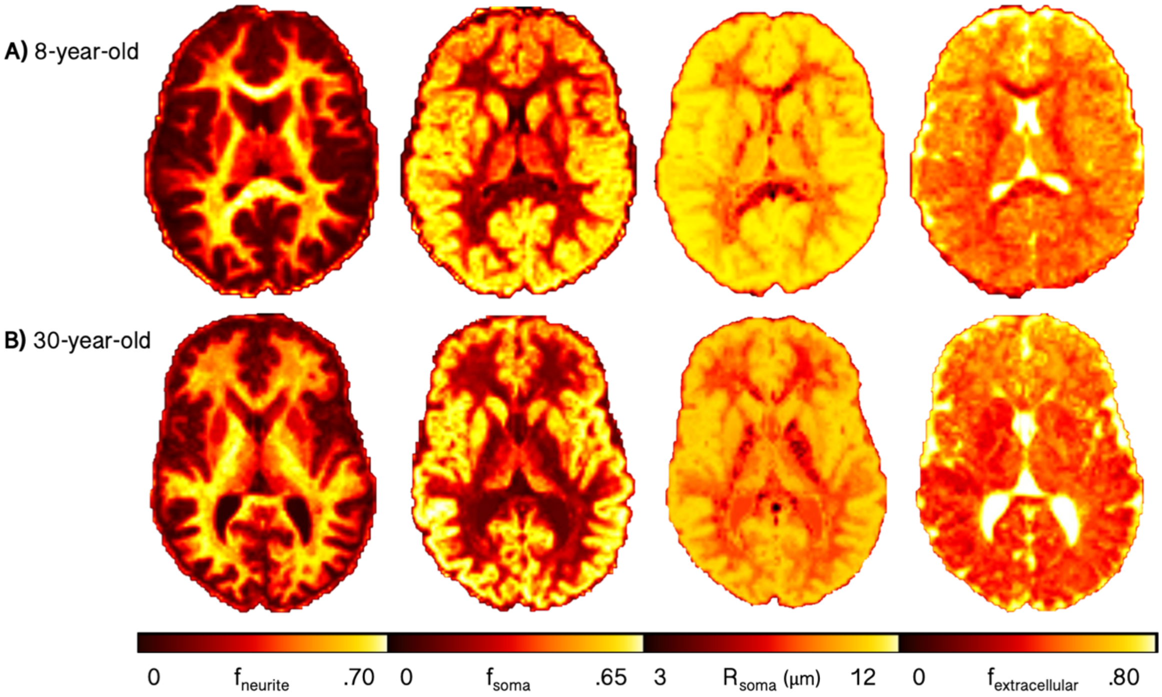

Figure S11: Representative maps of the four SANDI measures from two participants: A) an 8-year-old and B) a 30-year-old. Abbreviations: $f_{extracellular}$: extracellular signal fraction; $f_{neurite}$: neurite signal fraction; f_{soma}: soma signal fraction; R_{soma}: apparent soma radius, in μm.

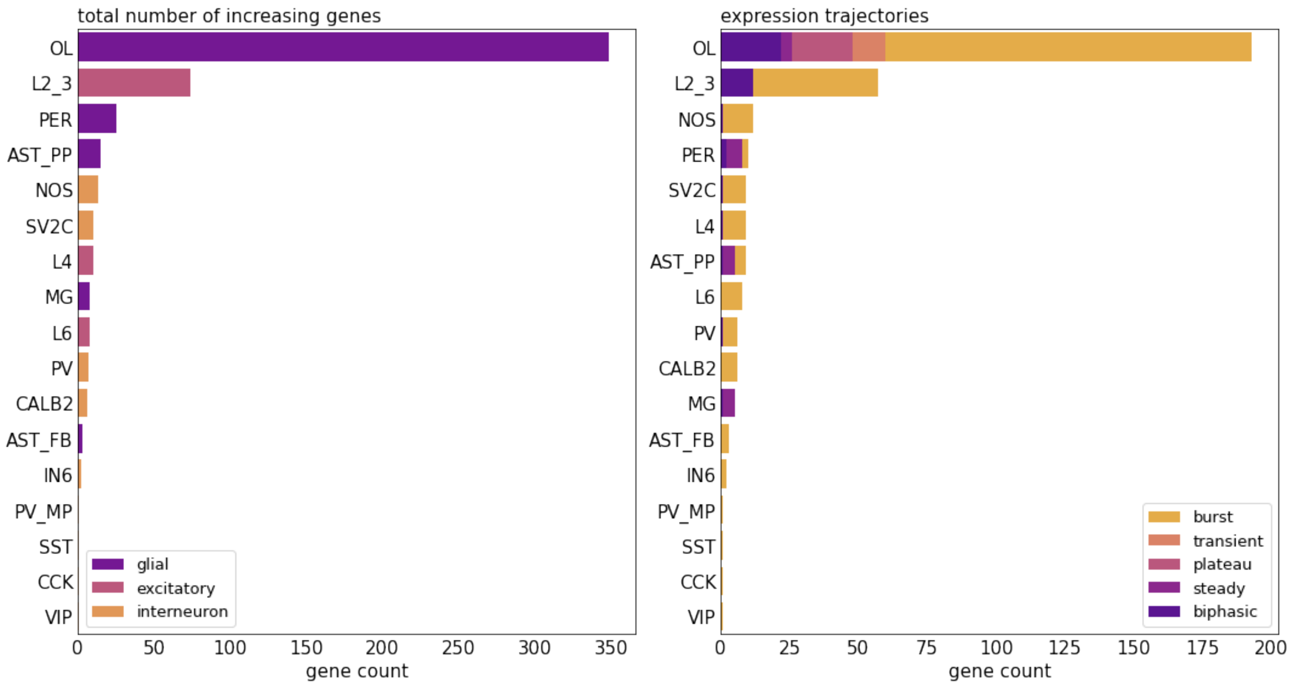

Figure S12: Validation of our gene expression findings in an independent single-cell RNA dataset[4]. Cell-specific genes that had an onset of expression in childhood (>4 years) followed by a rapid increase into adolescence and adulthood were mainly expressed by oligodendrocytes.

Tables

Table S1: Cortical regions sampled for gene expression analyses, and their corresponding Yeo7 network.

Abbreviation	Cortical region	PsychENCODE	BrainCloud	Yeo7 network
A1C	Primary auditory	+	-	Ventral attention
DLPFC	Dorsolateral pre-frontal	+	+	Frontoparietal
IPC	Inferior parietal	+	-	Ventral attention
ITC	Inferior temporal	+	-	Limbic
M1	Primary motor	+	-	Somatomotor
MFC	Medial frontal	+	-	Default mode
OFC	Orbito-frontal	+	-	Limbic
S1	Primary somatosensory	+	-	Somatomotor
STC	Superior temporal	+	-	Default mode
V1	Primary visual	+	-	Visual
VLPFC	Ventrolateral pre-frontal	+	-	Ventral attention

Note: + indicates the region was sampled in the respective database. Far right column indicates the Yeo7 network overlapping the cortical region sampled in the gene expression analysis.

Table S2: Summary statistics for age-related patterns of MRI-derived morphology and microstructure. A simple linear regression model was fitted using the lm() function in R to examine the relationship between diffusion measures and age and sex. P-values reported are raw outputs and not corrected for multiple comparisons.

Network	Metric	Age				Sex				R^2
		Coeff	Lower	Upper	p	Coeff	Lower	Upper	p	
defaultmode	FA	-.47	-.66	-.29	**2.1E-06**	.39	.02	.76	.04	.29
defaultmode	MD (s/mm^2)	.06	-.15	.28	.57	.44	.01	.88	.04	.02
defaultmode	$f_{neurite}$	.77	.62	.92	**9.8E-17**	.32	.02	.61	.04	.55
defaultmode	f_{soma}	-.06	-.27	.16	.59	-.46	-.89	-.03	.04	.03
defaultmode	R_{soma} (µm)	-.73	-.88	-.57	**2.6E-14**	-.36	-.68	-.05	.02	.48
defaultmode	$f_{extracellular}$	-.31	-.52	-.11	**.003**	.29	-.12	.70	.16	.12
defaultmode	v_{ic}	.74	.59	.89	**3.3E-15**	.14	-.16	.45	.36	.51
defaultmode	ODI	.64	.47	.81	**2.7E-11**	-.16	-.49	.17	.34	.43
defaultmode	CTh (mm)	-.59	-.77	-.41	**7.8E-09**	-.28	-.64	.08	.13	.31
defaultmode	SA (mm^2)	-.04	-.24	.15	.64	1.00	.62	1.38	**1.2E-06**	.25
defaultmode	GMvol (mm^3)	-.37	-.55	-.20	**5.0E-05**	.82	.48	1.17	**8.8E-06**	.37
dorsalattention	FA	-.60	-.77	-.42	**1.1E-09**	.20	-.15	.54	.26	.38
dorsalattention	MD (s/mm^2)	.16	-.06	.37	.16	.31	-.12	.74	.16	.01
dorsalattention	$f_{neurite}$	.78	.64	.93	**1.0E-17**	.22	-.06	.51	.13	.57
dorsalattention	f_{soma}	-.35	-.56	-.15	**.001**	-.49	-.90	-.08	.02	.12
dorsalattention	R_{soma} (µm)	-.78	-.93	-.64	**2.0E-17**	-.35	-.64	-.06	.02	.56
dorsalattention	$f_{extracellular}$	-.16	-.37	.05	.14	.34	-.08	.77	.11	.05
dorsalattention	v_{ic}	.71	.55	.87	**1.6E-13**	.10	-.21	.42	.52	.47
dorsalattention	ODI	.71	.55	.86	**3.0E-14**	-.09	-.39	.22	.58	.51
dorsalattention	CTh (mm)	-.40	-.60	-.19	**2.4E-04**	-.15	-.56	.26	.48	.13
dorsalattention	SA (mm^2)	-.10	-.30	.11	.36	.69	.29	1.10	**1.1E-03**	.13
dorsalattention	GMvol (mm^3)	-.34	-.54	-.15	**5.7E-04**	.60	.22	.98	**2.4E-03**	.24
frontoparietal	FA	-.47	-.66	-.29	**2.4E-06**	.35	-.02	.72	.06	.28
frontoparietal	MD (s/mm^2)	.16	-.06	.37	.15	.42	-.01	.85	.06	.03
frontoparietal	$f_{neurite}$	.66	.48	.83	**3.3E-11**	.20	-.14	.54	.24	.39
frontoparietal	f_{soma}	-.08	-.30	.14	.47	-.41	-.84	.02	.06	.02
frontoparietal	R_{soma} (µm)	-.63	-.80	-.45	**4.4E-10**	-.35	-.70	.00	.05	.35
frontoparietal	$f_{extracellular}$	-.20	-.42	.01	.06	.29	-.13	.72	.17	.06
frontoparietal	v_{ic}	.59	.41	.77	**4.6E-09**	.01	-.35	.37	.96	.33
frontoparietal	ODI	.59	.42	.77	**1.7E-09**	-.18	-.53	.17	.30	.37
frontoparietal	CTh (mm)	-.29	-.50	-.08	**8.3E-03**	.07	-.35	.49	.73	.07
frontoparietal	SA (mm^2)	.01	-.20	.21	.96	.84	.44	1.24	**8.1E-05**	.16
frontoparietal	GMvol (mm^3)	-.20	-.40	.00	.05	.71	.31	1.10	**6.3E-04**	.18
limbic	FA	.04	-.18	.26	.72	.42	-.02	.85	.06	.02
limbic	MD (s/mm^2)	-.42	-.62	-.22	**8.8E-05**	.01	-.39	.41	.96	.16
limbic	$f_{neurite}$	.81	.67	.95	**2.0E-19**	.26	-.01	.53	.06	.61
limbic	f_{soma}	-.35	-.55	-.14	**.001**	-.06	-.48	.35	.76	.09
limbic	R_{soma} (µm)	-.75	-.90	-.60	**4.7E-16**	-.08	-.38	.22	.60	.54
limbic	$f_{extracellular}$	-.49	-.68	-.29	**3.7E-06**	-.13	-.52	.26	.51	.21
limbic	v_{ic}	.80	.67	.94	**3.5E-19**	.26	-.02	.53	.07	.60
limbic	ODI	.45	.25	.65	**1.9E-05**	-.11	-.50	.29	.59	.20
limbic	CTh (mm)	-.30	-.52	-.09	**5.5E-03**	-.24	-.66	.18	.26	.07
limbic	SA (mm^2)	.19	-.01	.39	.07	.84	.44	1.25	**6.9E-05**	.16
limbic	GMvol (mm^3)	-.04	-.25	.17	.73	.62	.20	1.04	**4.3E-03**	.08
somatomotor	FA	-.57	-.75	-.39	**1.4E-08**	.10	-.26	.46	.58	.33
somatomotor	MD (s/mm^2)	.34	.13	.55	**.002**	.39	-.02	.81	.06	.10
somatomotor	$f_{neurite}$	.73	.57	.88	**1.2E-14**	.14	-.17	.45	.38	.50
somatomotor	f_{soma}	-.50	-.69	-.31	**1.7E-06**	-.46	-.84	-.07	.02	.23
somatomotor	R_{soma} (µm)	-.71	-.87	-.54	**2.6E-13**	-.29	-.61	.03	.08	.46
somatomotor	$f_{extracellular}$	.09	-.13	.31	.42	.43	.00	.86	.05	.02
somatomotor	v_{ic}	.63	.46	.81	**1.6E-10**	.03	-.31	.37	.86	.38
somatomotor	ODI	.69	.52	.85	**8.9E-13**	.05	-.27	.38	.74	.45

somatomotor	CTh (mm)	-.40	-.60	-.19	**2.1E-04**	-.31	-.72	.10	.13	.13
somatomotor	SA (mm^2)	.00	-.19	.20	.97	.90	.51	1.29	**1.9E-05**	.19
somatomotor	GMvol (mm^3)	-.19	-.39	.01	.06	.64	.24	1.04	**2.2E-03**	.15
ventralattention	FA	-.52	-.71	-.34	**3.0E-07**	.10	-.27	.47	.59	.27
ventralattention	MD (s/mm^2)	.15	-.06	.37	.16	.44	.01	.87	.04	.03
ventralattention	$f_{neurite}$	.72	.57	.88	**3.3E-14**	.27	-.04	.59	.09	.48
ventralattention	f_{soma}	-.09	-.31	.13	.41	-.46	-.89	-.03	.04	.03
ventralattention	R_{soma} (μm)	-.70	-.87	-.54	**3.1E-13**	-.33	-.65	-.01	.05	.45
ventralattention	$f_{extracellular}$	-.22	-.43	-.01	.04	.35	-.07	.77	.10	.08
ventralattention	v_{ic}	.71	.55	.86	**8.5E-14**	.01	-.30	.33	.93	.48
ventralattention	ODI	.63	.46	.80	**1.6E-10**	.02	-.33	.36	.93	.38
ventralattention	CTh (mm)	-.09	-.31	.13	.42	.07	-.37	.51	.76	-.01
ventralattention	SA (mm^2)	.12	-.07	.31	.20	1.08	.70	1.45	**1.8E-07**	.26
ventralattention	GMvol (mm^3)	-.01	-.20	.18	.94	1.05	.68	1.43	**2.9E-07**	.26
visual	FA	-.42	-.60	-.23	**2.1E-05**	.55	.18	.92	**4.0E-03**	.29
visual	MD (s/mm^2)	-.13	-.35	.09	.25	.09	-.35	.52	.70	.00
visual	$f_{neurite}$	.77	.62	.92	**8.8E-17**	.33	.04	.63	.03	.55
visual	f_{soma}	-.24	-.45	-.02	.03	-.34	-.77	.08	.11	.04
visual	R_{soma} (μm)	-.65	-.82	-.48	**3.4E-11**	-.57	-.91	-.24	**1.1E-03**	.40
visual	$f_{extracellular}$	-.33	-.54	-.12	**.002**	.05	-.37	.46	.83	.09
visual	v_{ic}	.68	.52	.85	**2.5E-12**	.44	.11	.77	.009	.43
visual	ODI	.69	.53	.84	**1.3E-13**	-.17	-.48	.14	.29	.50
visual	CTh (mm)	-.61	-.78	-.43	**1.1E-09**	.00	-.35	.35	.99	.36
visual	SA (mm^2)	.03	-.16	.23	.72	.96	.57	1.35	**4.9E-06**	.21
visual	GMvol (mm^3)	-.29	-.47	-.11	**.002**	.90	.55	1.26	**2.5E-06**	.34

Note: Bold values indicate $p<.005$. Abbreviations: CTh: Cortical thickness; FA: fractional anisotropy; $f_{extracellular}$: extracellular signal fraction; $f_{neurite}$: neurite signal fraction; f_{soma}: soma signal fraction; GMvol: Grey matter volume; md: mean diffusivity; odi: orientation dispersion index; R_{soma}: apparent soma radius, in μm; SA: surface area; vic: intracellular volume fraction.

Table S3: Results of specific expression analysis across brain regions and development.

Developmental period	Brain region					
	Amygdala	Cerebellum	Cortex	Hippocampus	Striatum	Thalamus
Fetal						
Early	0.442	0.076	0.872	0.607	0.802	0.379
Early mid	0.797	0.943	**0.002***	0.654	0.54	**0.003***
Late mid	**0.035**	0.296	**5.860e-04***	0.411	0.987	0.776
Late	**5.022e-05***	0.08	**3.339e-06***	0.243	0.942	0.072
Infancy						
Early	0.058	0.186	0.102	0.104	0.096	**1.194e-05***
Late	0.081	0.926	0.296	**0.038**	0.076	0.058
Childhood						
Early	0.096	0.134	**0.007***	**0.003***	**1.781e-05***	**0.007***
Mid-late	**6.407e-05***	**0.005***	**0.015***	**0.002***	0.724	**3.037e-09***
Adolescence	**1.083e-09***	**6.192e-04***	**8.306e-13***	**8.253e-06***	**2.780e-13***	**6.921e-15***
Young Adulthood	**1.257e-09***	**5.167e-04***	**1.676e-24***	**5.041e-06***	**5.563e-09***	**6.453e-18***

Note: Bold values indicate p<.05. * indicates p<.05 following Benjamini-Hochberg correction. To evaluate the significance of overlap between a candidate gene list and the list of transcripts enriched in a particular cell type, we used the Fisher's exact test[5] with Benjamini–Hochberg multiple testing correction[5] (p<.05).

Supplemental Information

Gene list

Below is our gene-list, with genes included if they had a significantly positive association with age in both the BrainCloud and PsychENCODE datasets (N=467).

AATK, ABCC4, ABCC8, ABCD2, ABLIM2, ADORA2B, AFAP1, AKAP13, ALCAM, ANKH, ANKRD40, ANO5, APC, APOE, ARHGAP15, ARHGAP25, ARHGAP26, ARHGEF25, ARL6IP1, ARL6IP5, ARPC1B, ASH1L, ASPA, ASTN2, ATF6, ATP10A, ATP6AP1, ATP6V0B, ATP6V1E1, ATPAF1, AUH, B3GNT5, B4GALT1, BACH2, BCAN, BCAS1, BCAS2, BCHE, BCL2L2, BEX4, BHLHE40, BICD1, BLM, BTG3, BTNL9, C1QA, CABP1, CACNG2, CADM1, CAMK1D, CAMK2G, CAMKK1, CAP1, CAPN3, CAPNS1, CARNS1, CASK, CCBE1, CCDC50, CD163, CD300A, CD83, CD84, CD93, CDC37, CDK14, CDKN1C, CEND1, CERCAM, CFL2, CHGB, CHRM2, CHST11, CIT, CKS2, CLDND1, CLIC1, CLIP4, CLMN, CMTM3, CMTM7, CNDP1, CNGB1, CNTNAP4, COL19A1, COL24A1, CORO2B, CORO6, CPLX1, CREM, CRMP1, CRYAB, CSPG5, CTNNA2, CX3CL1, CXCL1, CYFIP1, CYSLTR1, CYTH1, DAB1, DAB2, DAPK1, DDX24, DDX51, DIAPH2, DIO2, DKK3, DLGAP4, DNAH6, DNAJC12, DNM1, DOCK5, DOK5, DPYD, DPYSL3, EDEM1, EDN3, EFS, EGR1, EGR2, EIF4A3, ELMO1, ENO2, ENPP5, EPB41L1, EPB41L3, EPHB1, EPHB6, ERMN, ETF1, ETS2, ETV5, ETV6, EVI2A, EXOC4, EXT1, EXTL2, FADS2, FAIM2, FAM110B, FAM13A, FAM153B, FAM168B, FAM171A1, FAM171B, FAM81A, FARP1, FCGRT, FLCN, FRZB, FSTL4, FUT9, GABBR1, GABRA2, GABRG1, GABRG2, GFOD1, GGA2, GGT5, GHITM, GLA, GLDN, GLS, GNA14, GNAS, GNG4, GNPTAB, GPD2, GPM6A, GPR155, GPX3, GRIA3, GRIK4, GSG1L, HAPLN2, HAS2, HAVCR2, HCK, HES1, HHATL, HLA-A, HMOX1, HPGDS, HS2ST1, HSD11B1, HSPA1A, HSPB8, ID3, IGF1, IKBIP, IL1RAP, ILDR2, INHBA, INPP5D, INPP5F, IQCA1, IQGAP2, IQSEC1, ITPR1, KCNAB2, KCNAB3, KCNC1, KCNH8, KCNIP2, KCNJ16, KCNJ8, KCTD12, KDR, KIAA0319, KIAA0513, KIAA1324, KIF21A, KIF5A, KLF12, KLF6, KLK6, LAIR1, LAMA4, LAMP5, LANCL1, LAPTM5, LCP1, LDB3, LEF1, LGALS1, LGALS9, LHFPL2, LIMS1, LIMS2, LPAR6, LPCAT1, LPCAT4, LPIN1, LRMP, LRRC3B, LUZP2, LY86, LYN, LYPD1, MAGI3, MAOB, MAP1LC3A, MAP3K13, MAP6D1, MAP7, MAP7D2, MARCKS, MCF2L2, MCL1, MEIS2, MEIS3, MFSD6, MGAT4C, MID1, MPDZ, MPHOSPH8, MPP1, MYCT1, NAGA, NAMPT, NAP1L2, NAP1L3, NAPEPLD, NCDN, NCKAP1L, NECAB1, NEFL, NEFM, NFIB, NINJ2, NIPAL3, NISCH, NKTR, NLGN1, NR3C2, NRXN2, NTM, NTN4, NTNG2, OAS2, OLFM2, OLFML3, OPALIN, OSBP2, OXR1, PABPC1, PAG1, PAIP2, PALMD, PAM, PARVG, PCDH10, PCDH11X, PCDH11Y, PCDH15, PDE8A, PDGFRA, PDPN, PDZRN3, PEBP1, PECAM1, PELI1, PENK, PFKP, PFN1, PHF20L1, PHYHIP, PI4KA, PIK3R1, PIP4K2A, PKD1, PLAUR, PLCG2, PLEKHA1, PLEKHA6, PLEKHH1, PLS3, POLR2F, POLR2L, PPFIBP1, PPP2R1A, PPP3CA, PRICKLE2, PRKCH, PRSS35, PRUNE2, PTAR1, PTK2B, PTPN5, PTPN6, PTPRG, PTPRK, PTPRM, PTPRT, PXK, PYGO1, QDPR, RAB20, RAB32, RALGPS2, RANGAP1, RAP2B, RAPGEF2, RASA1, RASGEF1B, RASGRF2, RB1, RBP7, RCAN2, RGR, RHOC, RHPN2, RILPL2, RIMS3, RIPK2, ROBO1, RPL13, RPL13A, RPL18, RPL27A, RPL35A, RPL36, RPL38, RPL8, RPS10, RPS16, RPS18, RPS23, RPS28, RPS3, RPS6KA2, RPS6KC1, S100A10, S100A6, S1PR5, SCN1A, SCN1B, SDC3, SDK1, SEMA5A, SERPINE2, SFMBT2, SGCZ, SH3GL2, SH3RF3, SHROOM3, SIPA1L1, SLA, SLC24A3, SLC2A5, SLC35F1, SLC39A8, SLC5A11, SNAP25, SNCG, SORBS1, SORL1, SORT1, SPARCL1, SPP1, SPRY2, SRR, SRRM2, SRRM4, SRSF5, ST18, ST8SIA4, STAMBPL1, SVIP, SYNJ2, SYT12, TAGLN2, TAL1, TBK1, TBXAS1, TCEAL2, TCFL5, TCN2, TFRC, THSD7A, THSD7B, TIE1, TLR7, TMEM130, TMEM156, TMEM56, TNFRSF25, TNNT2, TNRC6A, TOMM20, TOR3A, TOX, TPPP, TPT1, TRANK1, TRIB1, TSPAN14, TSPAN5, TSPYL2, TTBK2, TTL, TTLL11, TUBB, TUBB2B, UBAC2, UBE2F, UCP2, UGDH, UNC80, UPP1, USP25, UST, VAMP8, VSNL1, VWA5B2, WBP2, WIPF3, WLS, WSB1, WWC1, XKR4, XKR6, YPEL3, YWHAE, YWHAH, ZC3HAV1, ZMAT1

Age prediction of microstructural measures in the visual network

Using an age-prediction random forest model for each microstructural measure in the visual network, we found that R_{soma} provided the most accurate age-prediction (cross-validated R^2= .58), followed by $f_{neurite}$ (R^2= .56), and f_{soma} (R^2=.28). Model fitting did not converge for $f_{extracellular}$. NODDI measures showed R^2_{odi}=.46, and R^2_{vic}=.36. Feature importance analysis revealed that association cortices within the visual network had the highest contribution (top 5%) to age prediction (Fig S10b,c,d). Notably, region 31a (posterior cingulate cortex) consistently influenced age prediction across multiple measures, with R_{soma} contributing 63%, ODI 7% and v_{ic} 5.4%. Additional top-ranking regions included dorsal visual area, V3A (v_{ic} = 45%), lateral temporal area, TE2a (v_{ic} = 17.8 %, $f_{neurite}$ = 5.1%), retrosplenial cortex, RSC, (vic=7.9%), auditory association area, A5 ($f_{neurite}$=5.5%), and lateral occipital area, LO3, (f_{soma}=5.4%). These regions (depicted in Fig S10c) represent cortical endpoints of developmentally sensitive tracts, identified through tractography, such as the posterior corpus callosum, cingulum, and inferior longitudinal fasciculus (Fig S10d).

References

1. Xu X, Wells AB, O'Brien DR, Nehorai A, Dougherty JD. Cell type-specific expression analysis to identify putative cellular mechanisms for neurogenetic disorders. J Neurosci. 2014;34(4):1420-31.
2. Keller D, Erö C, Markram H. Cell densities in the mouse brain: a systematic review. Frontiers in neuroanatomy. 2018;12:83.
3. Glasser MF, Coalson TS, Robinson EC, Hacker CD, Harwell J, Yacoub E, et al. A multi-modal parcellation of human cerebral cortex. Nature. 2016;536(7615):171-8.
4. Velmeshev D, Perez Y, Yan Z, Valencia JE, Castaneda-Castellanos DR, Wang L, et al. Single-cell analysis of prenatal and postnatal human cortical development. Science. 2023;382(6667):eadf0834.
5. Fisher RA. On the mathematical foundations of theoretical statistics. Philosophical transactions of the Royal Society of London Series A, containing papers of a mathematical or physical character. 1922;222(594-604):309-68.
