# Supplementary material for: MRI signatures of cortical microstructure in human development align with oligodendrocyte cell-type expression
Source: Nat Commun. 2025 Apr 7;16:3317. doi: 10.1038/s41467-025-58604-w (PMC11977195; doi:10.1038/s41467-025-58604-w)
Supplement: Supplementary file 2 — Description of additional supplementary file [file 41467_2025_58604_MOESM2_ESM.pdf]

Description of Additional Supplementary File

Supplementary code 1 - code for gene expression analysis
