# Supplementary material for: MRI signatures of cortical microstructure in human development align with oligodendrocyte cell-type expression
Source: Nat Commun. 2025 Apr 7;16:3317. doi: 10.1038/s41467-025-58604-w (PMC11977195; doi:10.1038/s41467-025-58604-w)
Supplement: Supplementary file 4 — Reporting Summary [file 41467_2025_58604_MOESM4_ESM.pdf]

nature portfolio

Corresponding author(s): Sila Genc

Last updated by author(s): Mar 20, 2025

Reporting Summary

Nature Portfolio wishes to improve the reproducibility of the work that we publish. This form provides structure for consistency and transparency in reporting. For further information on Nature Portfolio policies, see our Editorial Policies and the Editorial Policy Checklist.

Statistics

For all statistical analyses, confirm that the following items are present in the figure legend, table legend, main text, or Methods section.

n/a	Confirmed	
☐	☒	The exact sample size (*n*) for each experimental group/condition, given as a discrete number and unit of measurement
☐	☒	A statement on whether measurements were taken from distinct samples or whether the same sample was measured repeatedly
☐	☒	The statistical test(s) used AND whether they are one- or two-sided<br>*Only common tests should be described solely by name; describe more complex techniques in the Methods section.*
☐	☒	A description of all covariates tested
☐	☒	A description of any assumptions or corrections, such as tests of normality and adjustment for multiple comparisons
☐	☒	A full description of the statistical parameters including central tendency (e.g. means) or other basic estimates (e.g. regression coefficient) AND variation (e.g. standard deviation) or associated estimates of uncertainty (e.g. confidence intervals)
☐	☒	For null hypothesis testing, the test statistic (e.g. *F*, *t*, *r*) with confidence intervals, effect sizes, degrees of freedom and *P* value noted<br>*Give P values as exact values whenever suitable.*
☒	☐	For Bayesian analysis, information on the choice of priors and Markov chain Monte Carlo settings
☐	☒	For hierarchical and complex designs, identification of the appropriate level for tests and full reporting of outcomes
☒	☐	Estimates of effect sizes (e.g. Cohen's *d*, Pearson's *r*), indicating how they were calculated

Our web collection on statistics for biologists contains articles on many of the points above.

Software and code

Policy information about availability of computer code

Data collection	REDCap was used for parent and child survey data collection.
Data analysis	MRI data pre-processing and analysis was performed using the following software packages: MRtrix3; FSL; MATLAB; ANTs; FreeSurfer; RStudio; Python. Code to perform the gene expression analyses are provided as R scripts.

For manuscripts utilizing custom algorithms or software that are central to the research but not yet described in published literature, software must be made available to editors and reviewers. We strongly encourage code deposition in a community repository (e.g. GitHub). See the Nature Portfolio guidelines for submitting code & software for further information.

Data

Policy information about availability of data

All manuscripts must include a data availability statement. This statement should provide the following information, where applicable:

- Accession codes, unique identifiers, or web links for publicly available datasets
- A description of any restrictions on data availability
- For clinical datasets or third party data, please ensure that the statement adheres to our policy

Original datasets are accessible through the original publications, including the MICRA 82 neuroimaging repeatability dataset (osf.io/z3mkn/), PsychENCODE Human mRNA-seq processed data (Gene expression in RPKM: development.psychencode.org) and BrainCloud (https://www.ncbi.nlm.nih.gov/geo/query/acc.cgi?acc=GSE30272). Source data to generate figures from openly available data are provided with this paper. Due to the inclusion of minors (under 18 participants) in

the MRI portion of our study, the availability of derived or identifiable data from our participant cohort is restricted due to privacy concerns. Derived data supporting the findings of the imaging analyses are available by contacting the corresponding author in writing via email (Dr Sila Genc: gencs@cardiff.ac.uk), allowing four weeks for access requests to be granted.

Research involving human participants, their data, or biological material

Policy information about studies with human participants or human data. See also policy information about sex, gender (identity/presentation), and sexual orientation and race, ethnicity and racism.

Reporting on sex and gender	Our results report on sex, not gender. We administered a parent survey to all children and adolescents enrolled in the study (participants aged 8-19 years), as well as a modified version of the survey to participants themselves aged 11-19 years. Included in the survey was the Pubertal Developmental Scale (PDS) which asks questions specific to female and male sex characteristics and pubertal progression.
Reporting on race, ethnicity, or other socially relevant groupings	We did not define or categorize participants based on their race, ethnicity or other socially relevant groupings. We collected information on socio-economic status (e.g., parental education, parental income) in our study but did not report this in the current manuscript. This information can be reported upon request.
Population characteristics	We included age and sex as covariates in the majority of analyses. Select follow-up analyses also investigated the impact of pubertal stage on age relationships.
Recruitment	Participants that underwent MRI were recruited from the local community via public outreach events in Cardiff, Wales, United Kingdom.
Ethics oversight	The Cardiff University School of Psychology ethics committee.

Note that full information on the approval of the study protocol must also be provided in the manuscript.

Field-specific reporting

Please select the one below that is the best fit for your research. If you are not sure, read the appropriate sections before making your selection.

☒ Life sciences ☐ Behavioural & social sciences ☐ Ecological, evolutionary & environmental sciences

For a reference copy of the document with all sections, see nature.com/documents/nr-reporting-summary-flat.pdf

Life sciences study design

All studies must disclose on these points even when the disclosure is negative.

Sample size	We included a large paediatric cohort (n=88) to address our research questions.
Data exclusions	We excluded 4 MRI datasets due to motion artefact identified on T1-weighted imaging.
Replication	We assessed the repeatability of microstructural measures in the cortex in six healthy adults scanned 5 times within 2 weeks using the MICRA cohort (Koller, NeuroImage, 2021) and showed very high repeatability of SANDI measures. We have provided these data in source data (Fig 2d)
Randomization	Randomization was not relevant as this study did not compare interventions or groups. Age and sex were included as covariates of interest in imaging-based analyses.
Blinding	Blinding was not relevant as we were primarily studying continuous relationships of imaging-derived measures with age.

Reporting for specific materials, systems and methods

We require information from authors about some types of materials, experimental systems and methods used in many studies. Here, indicate whether each material, system or method listed is relevant to your study. If you are not sure if a list item applies to your research, read the appropriate section before selecting a response.

Materials & experimental systems

n/a	Involved in the study
☒	☐ Antibodies
☒	☐ Eukaryotic cell lines
☒	☐ Palaeontology and archaeology
☒	☐ Animals and other organisms
☒	☐ Clinical data
☒	☐ Dual use research of concern
☒	☐ Plants

Methods

n/a	Involved in the study
☒	☐ ChIP-seq
☒	☐ Flow cytometry
☐	☒ MRI-based neuroimaging

Plants

Seed stocks

Report on the source of all seed stocks or other plant material used. If applicable, state the seed stock centre and catalogue number. If plant specimens were collected from the field, describe the collection location, date and sampling procedures.

Novel plant genotypes

Describe the methods by which all novel plant genotypes were produced. This includes those generated by transgenic approaches, gene editing, chemical/radiation-based mutagenesis and hybridization. For transgenic lines, describe the transformation method, the number of independent lines analyzed and the generation upon which experiments were performed. For gene-edited lines, describe the editor used, the endogenous sequence targeted for editing, the targeting guide RNA sequence (if applicable) and how the editor was applied.

Authentication

Describe any authentication procedures for each seed stock used or novel genotype generated. Describe any experiments used to assess the effect of a mutation and, where applicable, how potential secondary effects (e.g. second site T-DNA insertions, mosiacism, off-target gene editing) were examined.

Magnetic resonance imaging

Experimental design

Design type: No functional tasks were administered.

Design specifications: No functional tasks were administered.

Behavioral performance measures: No functional tasks were administered.

Acquisition

Imaging type(s): Structural T1, Multi-shell diffusion-weighted imaging.

Field strength: 3 Tesla (with 300 mT/m maximum amplitude field gradients).

Sequence & imaging parameters

T1-weighted anatomical images were acquired using a 3D Magnetization Prepared Rapid Gradient Echo (MP-RAGE) sequence with the following parameters: TE=2 ms, TR=2300 ms, TI=857 ms, and flip angle of 9°. The field of view was 256×256, the imaging matrix 256×256, with 192 slices of 1 mm slice thickness.

Multi-shell dMRI data were acquired using a Stejskal-Tanner EPI sequence with an anterior-to-posterior phase-encoding direction. Images covered the whole brain with field of view 220×220 mm, imaging matrix 110×110, and 66 slices of 2 mm slice thickness. TE=59 ms, TR=3000 ms, Δ = 23.3 ms, δ=7 ms. Data were acquired at five different b-values: b=500, 1200, 2400, 4000, and 6000 s/mm2; with 30 and 60 non-collinear directions used for b≤1200 s/mm2 and b≥2400 s/mm2, respectively. In addition, 14 non-diffusion weighted images were distributed uniformly throughout the protocol. One additional volume was acquired with reversed phase encoding for the purpose of EPI distortion correction.

Area of acquisition: Whole brain scan

Diffusion MRI ☒ Used ☐ Not used

Parameters: dMRI data were acquired at five different b-values: b=500, 1200, 2400, 4000, and 6000 s/mm2; with 30 and 60 non-collinear directions used for b≤1200 s/mm2 and b≥2400 s/mm2, respectively and 14 non-diffusion weighted images were distributed uniformly throughout the protocol. No cardiac gating was used.

Preprocessing

Preprocessing software: MRtrix3 (v3, 27.02.2020), FSL (v6.0.5), ANTS (v2.1.0), FreeSurfer (v6.0)

Normalization: Data were not normalized as all analyses were performed in individual subject space. A template was used to extract 7 functionally defined networks (Yeo et al., J Neurophysiol, 2011) and linearly transformed to individual subject (T1-weighted)

space using FLIRT (FSL).

Normalization template: The Yeo_JNeurophysiol11_MNI152 atlas was used to obtain cortical networks, but quantitative diffusion measures were extracted in individual subject space.

Noise and artifact removal: dMRI data underwent pre-processing to remove thermal noise (using MPPCA), gibbs ringing artefact, signal drift, and eddy-current, motion and susceptibility-induced distortions.

Volume censoring: We did not exclude any volumes within individual datasets.

Statistical modeling & inference

Model type and settings: Mixture of general linear models and general additive models.

Effect(s) tested: Statistics were used to test age relationships of microstructural MRI data and gene expression data.

Specify type of analysis: ☐ Whole brain ☐ ROI-based ☒ Both

Anatomical location(s): The Yeo_JNeurophysiol11_MNI152 atlas was used to obtain seven functionally-defined cortical networks.

Statistic type for inference (See Eklund et al. 2016): Voxel-averaging within cortical networks.

Correction: Stringent p-value thresholding at p<.005, as per recommendations from Benjamin et al., Nat Hum Behaviour, 2018.

Models & analysis

n/a	Involved in the study	
☒	☐	Functional and/or effective connectivity
☒	☐	Graph analysis
☐	☒	Multivariate modeling or predictive analysis

Multivariate modeling and predictive analysis: To identify important regions that contribute to age-related differences in all the studied microstructural measures, we performed age-prediction using a random forest regressor (5-fold cross-validation) for age prediction with PyCaret (www.pycaret.org). For each microstructural measure, we randomly split the data into training and validation sets using an 80-20 ratio (total N=88: 70 training; 18 testing). Then, we performed feature scaling to ensure that all input variables were on a similar scale prior to model fitting. The performance of the model was evaluated on the validation dataset. Finally, the features with the largest weight coefficients were extracted to identify specific cortical regions where variance in cortical microstructure was associated with age-related changes.
